# A novel cytosolic NADH:quinone oxidoreductase from *Methanothermobacter marburgensis*

**DOI:** 10.1042/BSR20140143

**Published:** 2014-12-23

**Authors:** Eva Ullmann, Tien Chye Tan, Thomas Gundinger, Christoph Herwig, Christina Divne, Oliver Spadiut

**Affiliations:** *Vienna University of Technology, Institute of Chemical Engineering, Research Area Biochemical Engineering, Vienna, Austria; †School of Biotechnology, KTH Royal Institute of Technology, Albanova University Center, Roslagstullsbacken 21, S-10691 Stockholm, Sweden; ‡Department of Medical Biochemistry and Biophysics, Karolinska Institutet, Scheelelaboratoriet, Scheeles väg 2, S-17177 Stockholm, Sweden

**Keywords:** crystal structure, cytoplasm, *Methanothermobacter marburgensis*, NADH regeneration, NADH:quinone oxidoreductase, acetyl-CoA, acetyl coenzyme A, CER, CO_2_ evolution rate, CV, column volume, DCPIP, 2,6-dichloroindophenol sodium salt hydrate, FMN, flavin mononucleotide, IPTG, isopropyl-β-D-thiogalactopyranoside, MdaB, modulator of drug activity B, PMF, proton motive force, r.m.s.d., root-mean-square deviation, TCA, tricarboxylic acid

## Abstract

*Methanothermobacter marburgensis* is a strictly anaerobic, thermophilic methanogenic archaeon that uses methanogenesis to convert H_2_ and CO_2_ to energy. *M. marburgensis* is one of the best-studied methanogens, and all genes required for methanogenic metabolism have been identified. Nonetheless, the present study describes a gene (Gene ID 9704440) coding for a putative NAD(P)H:quinone oxidoreductase that has not yet been identified as part of the metabolic machinery. The gene product, *Mm*NQO, was successfully expressed, purified and characterized biochemically, as well as structurally. *Mm*NQO was identified as a flavin-dependent NADH:quinone oxidoreductase with the capacity to oxidize NADH in the presence of a wide range of electron acceptors, whereas NADPH was oxidized with only three acceptors. The 1.50 Å crystal structure of *Mm*NQO features a homodimeric enzyme where each monomer comprises 196 residues folding into flavodoxin-like α/β domains with non-covalently bound FMN (flavin mononucleotide). The closest structural homologue is the modulator of drug activity B from *Streptococcus mutans* with 1.6 Å root-mean-square deviation on 161 Cα atoms and 28% amino-acid sequence identity. The low similarity at sequence and structural level suggests that *Mm*NQO is unique among NADH:quinone oxidoreductases characterized to date. Based on preliminary bioreactor experiments, *Mm*NQO could provide a useful tool to prevent overflow metabolism in applications that require cells with high energy demand.

## Abstract 2

A novel NADH:quinone oxidoreductase, *Mm*NQO, from *Methanothermobacter marburgensis* was identified. *Mm*NQO oxidizes NADH with several electron acceptors and is structurally similar to bacterial MdaB. It is localized in the cytosol and may provide a useful tool to prevent overflow metabolism.

## INTRODUCTION

Aerobic cellular respiration in eukaryotes involves glycolysis, TCA (tricarboxylic acid) cycle (also Krebs cycle or citric acid cycle) and respiratory chain. During glycolysis, glucose is converted to pyruvate, followed by oxidative decarboxylation to acetyl-CoA (acetyl coenzyme A). Acetyl-CoA enters the TCA cycle within the mitochondrial matrix where oxidation occurs concomitantly with reduction of NAD^+^ to NADH, and FAD to FADH_2_. The large quantities of NADH and FADH_2_ generated provide the high-potential reducing equivalents required for the ensuing electron-transfer events that build the PMF (proton motive force) necessary to power ATP synthesis through oxidative phosphorylation. This electron-transfer chain of inner mitochondrial-membrane protein complexes includes three proton pumps: NADH dehydrogenase (NADH:ubiqinone oxidoreductase; complex I [1]); cytochrome *bc_1_* reductase (ubiquinol:cytochrom *c* oxidoreductase; complex III); and cytochrome *c* oxidase (complex IV). Succinate dehydrogenase (succinate:ubiquinone oxidoreductase; complex II) is part of the TCA cycle, and does not pump protons, but rather serves as a link between TCA cycle and respiratory chain. The electrochemical proton gradient formed across mitochondrial membrane couples the respiratory chain to production of ATP through oxidative phosphorylation catalysed by ATP synthase (complex V).

In prokaryotes, the respiratory chain is confined to the cytoplasmic membrane in the intermembrane space. For *Escherichia coli*, the proton gradient at the membrane is generated by two types of enzymes, many of which have been characterized [2] (Supplementary Table S1): (i) dehydrogenases, which oxidize organic substrates and reduce ubiquinone; and (ii) oxidases, which oxidize ubiquinol and reduce molecular oxygen to water. Two membrane-bound *E. coli* NADH:ubiquinone oxidoreductases, NDH-1 and NDH-2, have been described [3–6], where NDH-1 operates as proton pump and shows sequence homology to the eukaryotic mitochondrial complex I, whereas NDH-2 is a single-subunit enzyme that catalyses the same reaction but without generating a PMF. Although NDH-2 is not directly involved in energy formation in *E. coli*, it performs an essential role by oxidizing excessive NADH to NAD^+^. If the rate of glucose consumption is higher than the respiratory capacity, the cells use pathways that are normally activated under anaerobic conditions to regenerate NAD^+^ and produce lactate or acetate, a phenomenon called overflow metabolism [7,8]. Since these undesired metabolites inhibit cell growth, it is desirable to avoid NADH accumulation [9].

The delicate NAD^+^/NADH balance has been studied in detail for *E. coli*, where it was demonstrated that increased amounts of NADH induced a metabolic shift towards fermentation even in the presence of oxygen excess [10]. In another study, the *nuo*-operon, coding for the NADH:ubiquinone oxidoreductase complex, was overexpressed in *E. coli* and increased activity with NADH and ferricyanide as electron acceptor was observed [11]. Unfortunately, the authors did not comment on the viability of the cells, or changes in metabolic activity. This is relevant since overexpression of wild-type NADH:ubiquinone oxidoreductases usually correlates with negative, sometimes detrimental effects for the cells, which have been rationalized by cellular stress associated with overexpression of integral membrane proteins [12].

Methanogenic archaea (methanogens) derive their energy from methanogenesis, a type of anaerobic respiration, where CO_2_ is reduced to methane in the presence of hydrogen. Based on their 16S rRNA, methanogens can be grouped into different classes, one being *Methanothermobacter* [13]. One of the most studied representatives of this class is *M. marburgensis* (formerly *M. thermoautotrophicum*) [13] for which the methanogenic pathway has been analysed in detail. The *M. marburgensis* genome has been sequenced (GenBank CP001710; [14]), and all enzymes essential for methanogenesis have been identified [15].

Interestingly, despite *M. marburgensis* being strictly anaerobic, we identified a novel gene coding for a yet uncharacterized NADH-dehydrogenase-like enzyme, i.e. gene ID 9704440 (UniProt D9PVS9). This was unexpected since all enzymes involved in energy metabolism of *M. marburgensis* have supposedly been identified. In the present study, the gene product of gene ID 9704440, here referred to as *Mm*NQO, was cloned, overexpressed in *E. coli* and characterized biochemically and structurally.

## EXPERIMENTAL

### Materials and reagents

Electron-acceptor substrates from Sigma Aldrich): coenzyme Q1, coenzyme Q10, DCPIP (2,6-dichloroindophenol sodium salt hydrate), potassium ferricyanide, 1,4-benzoquinone (1,4-BQ), ferrocenium hexafluorophosphate (Fc^+^), diethyl oxalacetate sodium salt, sodium fumarate dibasic, methyl red sodium (crystalline), 3,3′-methylene-bis(4-hydroxycoumarin). Electron acceptor substrates from Carl Roth (Germany): sodium thiosulphate anhydrous, DL-malic acid. Electron-donor substrates from Sigma Aldrich: β-nicotinamide adenine dinucleotide phosphate and reduced tetra (cyclohexylammonium) salt (NADPH).

### Sequence analyses

Sequence analyses for the three cloned *M. marburgensis* genes were performed with *BLAST* (http://blast.ncbi.nlm.nih.gov) [16] against existing sequence databases. The *TOPCONS* server (http://topcons.cbr.su.se) [17] was used to analyse the presence of transmembrane helices, and analysis of sequence motifs and domains was performed using the *SMART* server (http://smart.embl-heidelberg.de) [18].

### Preparation of genomic DNA and gene cloning

Genomic DNA from *M. marburgensis* was extracted with the peqGOLDTriFast kit (Peqlab). Frozen biomass of *M. marburgensis* was thawed and resuspended in 1.0 ml TriFast™ per 100 mg wet biomass. DNA concentration was measured using a Nanodrop device (ND_1000_ Spectrophotometer, Thermo Scientific). The gene 9704440 was amplified from genomic DNA using oligonucleotide primers listed in Supplementary Table S2 and cloned into a pET21a^+^ vector providing a C-terminal hexahistidine (His_6_) tag. PCRs were done in a total volume of 50 μl containing 2 μl dNTP mix (200 μM), 1 μl of each primer (50 μM), 10 μl 5× HF buffer, about 10 ng of genomic DNA and 0.5 μl Phusion Hot Start II Polymerase (2 units/μl, Finnzymes) in a S1000™ Thermal Cycler (Biorad) using the following program: 1 min at 98°C; 98°C 10 s—55°C 20 s–72°C 60 s (30 cycles); 72°C 10 min. The amplified PCR product was purified from the reaction mixture using a PCR purification kit (Qiagen), and cloned into the pET21a^+^ vector *via* the NheI and XhoI sites using standard cloning methods. The recombinant plasmid was transformed into *E. coli* BL21(DE3) by electroporation.

### Expression in shake flask and bioreactor cultures

#### Shake flasks

Shake flasks were used for the production of protein for biochemical and structural studies. A 10-ml aliquot of an overnight culture was transferred into 100 ml TB_amp_ medium in 1 litre baffled shake flasks and grown at 37°C with shaking (220 rpm). Induction at OD_600_ of about 0.6 was performed with 0.1 mM IPTG (isopropyl-β-D-thiogalactopyranoside) at 25°C. After 24 h, biomass was harvested by centrifugation (4,500 ***g***, 4°C, 20 min). *E. coli* BL21(DE3) harbouring the pET21a^+^ vector was included as negative control.

#### Bioreactor cultivations

For the pre-culture, fresh *E. coli* transformants were cultured in 100 ml DeLisa medium ([19]; Supplementary Table S3) in 1-litre baffled shake flasks at 37°C (220 rpm) for approximately 12 h. The grown pre-culture was transferred aseptically to the culture vessel. The inoculation volume was 10% of the final starting volume. Batch cultivations were carried out in a 3 l working volume bioreactor from Labfors. DeLisa medium was sterilized in the bioreactor and sterile glucose-monohydrate mixed with trace elements was added aseptically. The pH was adjusted to 7.4 using NH_4_OH solution (2.4–2.6 M), and 0.3 ml polypropylen glycol were added as anti-foam. The pO_2_ and pH levels were measured with sterile electrodes. Base consumption was determined gravimetrically. Cultivation temperature was set to 37°C and agitation was fixed to 1400 rpm. The culture was aerated with 2.0 vvm air and off-gas was measured. Recorded data were logged in a PIMS (process information management system; Lucullus, Securecell). After the complete consumption of the carbon source, as indicated by a drop in off-gas activity, the fed-batch was initiated.

For fed-batch cultivation, the feed with a glucose-monohydrate concentration of 440 g/l was aseptically added to the vessel. Using an in-house developed Kalman filter, the feed flow and specific growth rate (μ) was set to a value below the maximum specific growth rate (μ_max_) determined during the batch phase to prevent overflow metabolism. The two cultures of BL21(DE3) cells (one expressing 9704440 and a negative control) were induced with 0.1 mM IPTG after 7.5 h of fed-batch. Before IPTG was added, the temperature was decreased to 25°C to minimize the formation of inclusion bodies [20]. Samples were taken at the end of the batch, before induction, and at the end of cultivation. Dry cell weight was determined by centrifugation of 5 ml culture broth, washing the pellet with 5 ml deionized water und subsequent drying at 105°C to a constant weight in an oven. Substrate and metabolites in the supernatant were determined by HPLC analysis with an anion-exchange column (Supelcogel C-160H, Sigma Aldrich) equipped with a refractive index detector (Agilent Technologies). The flow rate of the mobile phase (0.1% H_3_PO_4_) was set to 0.5 ml/min and the system was run isocratically. Calibration for glucose, ethanol, acetate, citrate, malate, lactate, fumarate, oxaloacetate and pyruvate was done by measuring standards at concentrations of 0.1, 1.0 and 5.0 g/l, respectively.

### Protein purification and biochemical characterization

After harvesting, cells were resuspended in homogenization buffer (50 mM Mops, 500 mM NaCl, pH 7.4) to a final concentration of 20 g wet biomass per litre and protease inhibitor was added (two tablets of Complete EDTA-free protease inhibitor from Roche per 100 ml). Cells were homogenized using an Emulsiflex C3 homogenizer (Avestin). The homogenized suspension was centrifuged (20000 ***g***, 4°C, 15 min), and overall protein concentration and enzyme activity were measured. Soluble proteins and inclusion bodies were analysed by SDS–PAGE.

Purification of the 9704440 gene product was performed by IMAC (immobilized metal affinity chromatography). The protein solution was diafiltrated in binding buffer (20 mM Mops, 500 mM NaCl and 20 mM imidazole, pH 7.4) using a Centramate 500S system (PALL). A Ni^2+^-Sepharose 6 Fast Flow resin (GE Healthcare) resin was equilibrated with 5 CVs (column volumes) of binding buffer prior to sample loading. After loading, the column was washed with 5 CV of binding buffer, followed by elution of the target protein with a linear gradient (10 CV) of elution buffer (20 mM Mops, 500 mM NaCl, 500 mM imidazole, pH 7.4) and collection of 2.5-ml fractions. Sample binding and elution were performed at a flow rate of 60 cm/h, whereas equilibration and washing steps were done at 120 cm/h. Fractions containing the target protein were pooled, diafiltrated in 50 mM Mops (pH 7.4) and concentrated using Amicon Ultra-15 Centrifugal Filter Units with 10 kDa molecular weight cut-off (Merck-Millipore) to a final concentration of 3 mg/ml followed by purity analysis using SDS–PAGE and activity measurements. The protein content was measured spectrophotometrically at a wavelength of 595 nm according to the Bradford protocol using BSA as standard.

To identify the cofactor, the visible (VIS) spectrum was recorded in the range of 300–800 nm. Besides flavins, iron-sulphur clusters are found as prosthetic groups in NADH:quinone oxidoreductases with an absorption maximum at 455 nm [21]. The spectrum was recorded for 20 μl of purified 9704440 gene product using 50 mM MOPS buffer (pH 7.4) as blank. To analyse cofactor release, the enzyme was incubated with 5% trichloroacetic acid at 100°C for 2 h followed by centrifugation and repeating the measurement.

For measuring pH optimum and pH range, a 50 μl aliquot of enzyme solution was incubated with 50 μl of 100 mM buffer with the pH set to different pH values between 2.5 and 10.0 in steps of 0.5: citrate (2.5–5.5), carbonate (5.3–7.3), phosphate (6.2–8.2), Tris (7.5–9.0) and glycin (8.8–10.0). The enzyme was incubated at 30°C for 1 h and centrifuged at 20,000 ***g*** for 15 min to remove denaturated protein. Residual activity was measured in the supernatant using NADH and 1,4-BQ.

### Enzyme kinetics

Enzymatic activity of the 9704440 gene product was measured using NADH as electron donor with either coenzyme Q1 (ubiquinone) or 1,4-BQ as electron acceptor. 1 ml reaction mixture contained 50 mM MOPS (pH 7.4), 1 mM of electron acceptor and 1 mM of electron donor. The oxidation of NADH was followed at 340 nm (ε_340_=6.22 mM/cm). One unit of activity was defined as the amount of enzyme catalysing the oxidation of 1 μmol NADH/min. All measurements were done in duplicates. Catalytic constants for both NADH and NADPH were determined using a range of electron acceptors (see the Experimental section for details). Approximately 1 ml reaction mixture contained 50 mM Mops (pH 7.4), different concentrations of electron donor (1.0–150 μM) and a saturating concentration of 1 mM electron acceptor. The reaction was initiated by adding 20 μl purified enzyme at a concentration of 3 mg/ml. Measurements were done at 340 nm at 30°C in a UV-1601 spectrophotometer (Shimadzu) and the absorbance was recorded for 240 s using the UVPC Optional Kinetics software (Shimadzu). All measurements were done in duplicates. Catalytic constants were calculated using the program SigmaPlot.

### Crystallization and structure determination

Prior to crystallization, the buffer of the protein sample was exchanged by gel filtration to 20 mM Bis-Tris (pH 6.0), 150 mM NaCl and concentrated to 10 mg/ml. Crystallization screening was performed in Corning 3550 96-well sitting-drop plates using the sitting-drop vapour diffusion method, and 300 nl drops dispensed by a mosquito Crystal robotics (TTP Labtech) at protein-to-reservoir ratios of 1:1, 1:2 and 2:1. Well-formed crystals grew from a solution of 0.2 M NaCl, 0.1 M Bis-Tris (pH 5.5), and 25% PEG [poly(ethylene glycol)] 3350. Crystals were harvested from the mother liquor and vitrified in liquid nitrogen. X-ray intensity data to 1.50 Å resolution were collected at 100K at Diamond Light Source (UK) on macromolecular crystallography beamline *I*03, and the data processed (Supplementary Table S4) by the *XDS* package [22]. The resulting data indexed space group *P*2_1_, with cell parameters *a*=56.60 Å, *b*=94.69 Å, *c*=72.13 Å and *β*=93.34°.

Phases were obtained by molecular replacement with the *PHENIX* package [23] using the NAD(P)H:quinone oxidoreductase MdaB (modulator of drug activity B) from *Streptococcus mutans* as search model (PDB code 3LCM; [24]). An initial model was generated with *warpNtrace* included in the *ARP/wARP* package [25]. The model was refined at 1.50 Å resolution using *PHENIX* against the maximum-likelihood target and adjusted manually with the graphics softwares *COOT* [26] and *O* [27]. Refinement in *PHENIX* included XYZ-coordinate refinement, real-space refinement, and refinement of individual anisotropic temperature factors. The final model comprises four protein chains, A, B, C and D, forming two dimers (A/B and C/D), and four FMN molecules (one per monomer), and 427 water molecules. The gene construct cloned in the pET21d^+^ vector adds the sequence ^−2^MAS^0^ at the N-terminus and the sequence ^197^EHHHHHH^203^ corresponding to a non-cleavable hexahistidine tag at the C terminus. In the final model, each monomer is composed of residues 1-196 (UniProt D9PVS9). Additional residues from the cloning sequence modeled at the N-terminus include ^−1^AS^0^ for monomers A-D, and ^197^EHHHHHH^203^ at the C-terminus in monomers A and C. Monomer B contains the sequence ^197^EHHHH^201^ of the C-terminal tag, whereas monomer D retains only ^197^E of the tag. Residues not modeled due to lack of electron density: monomer A, 113–118; monomer B, 114–117, monomer C, 113–118, monomer D, 115–116. Structure-based fold similarity analyses were performed using the *DALI Lite* server (http://ekhidna.biocenter.helsinki.fi/dali_server) [28] and *PDBeFold* (http://www.ebi.ac.uk/msd-srv/ssm) [29]. Coordinates and structure factors have been deposited in the Protein Data Bank with accession code 4R81.

## RESULTS AND DISCUSSION

### Sequence analyses

The sequence of *M. marburgensis* gene ID 9704440 (UniProt D9PVS9) was analysed using *BLAST*, *SMART* and *TOPCONS*. Based on the results from these analyses, gene ID 9704440 codes for a soluble protein of 196 amino acids. The sequence shows similarity to a large number of bacterial NAD(P)H:quinone reductases with a varying degree of sequence identity in the range of 30–40%, of which several hits were loosely annotated as belonging to the bacterial MdaB.

### Expression in shake-flask and bioreactor cultures

Initially, the product for gene ID 9704440, hereafter referred to as *Mm*NQO, was expressed in shake flask cultures. To monitor production, intracellular protein samples were analysed for enzymatic activity and by SDS–PAGE. To optimize the amount of expressed *Mm*NQO, *E. coli* was cultivated in a bioreactor. Based on the data from the batch phase, the maximum specific growth rates (μ_max_) for both strains (one expressing 9704440 and a negative control) were calculated. The specific growth rate (μ) for each strain in the fed-batch phase was set below μ_max/2_ to avoid overflow metabolism [30]. Batch and fed-batch were performed at 37°C before temperature was decreased to 25°C upon induction. During induction, μ was set to only 10% of μ_max_ (Table 1). Off-gas was analysed online, and the respective CER (CO_2_ evolution rate), depicting metabolic activity, was calculated for both cultures of BL21(DE3), one containing the recombinant pET21a^+^ vector with the *Mm*NQO gene insert, and a negative control transformed with the empty pET21a^+^ (Supplementary Figure S1). Specific rates and yields were calculated from online and off-line data (Table 1). The batch phase for the two cultivations was similar, resulting in a similar amount of biomass. Nonetheless, the two cultures displayed different growth characteristics during the subsequent non-induced fed-batch resulting in different CER profiles and yields. For the negative control, more substrate was converted into CO_2_ than into biomass, whereas the opposite was observed for cells with *Mm*NQO (Table 1). Except for cells containing the recombinant plasmid in the induction phase, the carbon balance (C-balance) did not close to 1.0. However, we identified several metabolites in the cell-free cultivation broths (Supplementary Figures S2 and S3), and when taking these metabolites into account, it was possible to close the C-balance for the batch and induction phases of both cultures. For the other cultivation phases, the C-balances did not close to 1.0. As indicated by increasing concentrations of extracellular DNA and protein, this may be due to cell lysis. The observation that the apparent μ of the cells was different to the set μ during the non-induced fed-batch phase for both cultures may also be attributed to cell lysis since the Kalman filter, regulating the feed according to intact cells, did not respond to cell lysis.

**Table 1:** Cultivation of *E. coli* BL21(DE3) carrying empty plasmid and plasmid with *Mm*NQO insert

	pET21d^+^			pET21d^+^-*Mm*NQO		
	Batch	Fed-batch	Induction	Batch	Fed-batch	Induction
μ_max_ (h^−1^)	0.52	–	–	0.53	–	–
μ_set_ (h^−1^)	–	0.2	0.05	–	0.15	0.05
μ_real_ (h^−1^)	–	0.09	0.04	–	0.07	0.04
biomass (g/l)	5.1	20.9	44.8	5.2	16.2	35.6
*Y*_X/S_ (c-mol/c-mol)	0.33	0.34	0.24	0.33	0.47	0.44
*Y*_CO2/S_ (c-mol/c-mol)	0.44	0.40	0.34	0.44	0.35	0.53
C-balance without metabolites	0.77	0.74	0.58	0.77	0.82	0.97
**Metabolites (g/l)**						
Pyruvate	2.9	2.8	0.37	0.38	0.67	–
Lactate	–	0.49	0.73	0.52	0.71	0.52
Fumarate	–	–	0.32	–	–	–
Acetate	–	–	0.23	–	–	–
C-balance with metabolites	0.94	0.79	0.60	1.03	0.85	1.05

The observed discrepancy between the two cultures is interesting, especially the difference in metabolite formation (Supplementary Figures S2 and S3). Although a specific growth rate below μ_max/2_ was applied, overflow metabolism during the non-induced fed-batch phase resulted in the production of metabolites. This may be interpreted as a very low respiratory capacity of cells in both cultures. It is also interesting to note that pyruvate was the principal metabolite, and not acetate, indicating that the cells responded to an increased energy demand (accumulation of pyruvate for increased ATP production). Similar results have been observed by others, where metabolic shifts from anabolic to catabolic pathways occur after induction to allocate glucose for recombinant protein expression at the expense of biomass production [30].

After induction of cells transformed with the recombinant plasmid, no more metabolites were generated, and the previously formed metabolites were consumed (Table 1, Supplementary Figure S3). Thus, recombinant expression of *Mm*NQO appears to reduce overflow metabolism. This not only supports a function of *Mm*NQO as a NADH:quinone oxidoreductase, but also shows the potential of the enzyme to relieve overflow metabolism in recombinant *E. coli*. To date, detrimental overflow metabolism in *E. coli* is either tackled by tailoring the bioprocess, which requires extensive metabolomic analyses and the development of reliable soft sensor tools [31,32] or by engineering the *E. coli* strain. Current strain engineering strategies focus on modifying the substrate uptake system [33,34] and not on overexpression of NDH enzyme complexes, since previous studies have shown cellular stress associated with overexpression of integral membrane proteins [12]. Although cells with a reduced substrate uptake capacity are not extensively affected by overflow metabolism, these cells are physiologically impaired and are thus not useful for industrial production processes. Consequently, *E. coli* cells, where excessive NADH is regenerated by a cytosolic enzyme, such as *Mm*NQO, might describe an interesting host for energy demanding bioprocesses.

### Protein purification and biochemical characterization

Recombinant His_6_-tagged *Mm*NQO was purified to homogeneity, and the apparent molecular weight (*M*_w_), as determined with about 24 000 Da by SDS–PAGE, agreed well with the theoretical *M*_w_ of 23 538 Da. Any activity detected in the flow through during IMAC purification was ascribed to endogeneous *E. coli* NADH:quinone oxidoreductases. Purified *Mm*NQO was tested for NADH:quinone oxidoreductase activity using NADH or NADPH as electron donor and different electron acceptors (Supplementary Table S5). With NADH as electron donor, *Mm*NQO showed NADH:quinone oxidoreductase activity with all electron acceptors tested, whereas NADPH was only oxidized in the presence of three electron acceptors. The activity with NADPH as substrate was typically 2- to 20-fold lower compared with NADH. *Mm*NQO showed the highest specific activity with NADH and Ferrocenium hexafluorophosphate as electron acceptor.

FAD, FMN and sulphur–iron (Fe–S) clusters are common prosthetic groups in NADH:quinone oxidoreductases [2], where they typically show similar absorption maxima of 455 and 450 nm, respectively [21,35,36]. Purified *Mm*NQO displayed yellow colour and spectral properties with an absorption maximum at 455 nm (Supplementary Table S1 and Supplementary Figure S4). Incubation with trichloroacetic acid resulted in the disappearance of the 455 nm signal, suggesting that the cofactor is tightly bound to the protein.

### Enzyme kinetics

Michaelis–Mentens plots and kinetic constants were determined with either NADH (Table 2; Supplementary Figure S5) or NADPH (Table 3; Supplementary Figure S6) as electron donor using different electron acceptors. The apparent *K*_m_ values for NADH and NADPH vary from about 17 to 258 μM depending on the electron-acceptor used, which is in the range previously observed for prokaryotic NADH:quinone oxidoreductases [4,37,38]. The turn-over rates (*k*_cat_) fall in the range 4.95–19.8 min^−1^, with NADH/DCPIP being the most efficient redox couple. DCPIP is known to be a good electron acceptor for prokaryotic NADH:quinone oxidoreductases [4,39,40], and the wild-type *E. coli* NADH:quinone oxidoreductase reduces coenzyme Q1, DCPIP and ferricyanide using NADH as redox partner [40]. Furthermore, as for *Mm*NQO, *E. coli* NADH:quinone oxidoreductases display considerable variation in the kinetic constants depending on the electron acceptor used, as NDH-1 prefers ferricyanide as electron acceptor, whereas NDH-2, with higher affinity for NADH, prefers coenzyme Q1 [40]. With coenzyme Q1 as electron acceptor, *Mm*NQO shows a *K*_m_ value of 96.8 μM for NADH. This is more than 7-fold higher than the corresponding *K*_m_ value for *E. coli* NADH:quinone oxidoreductase (14 μM; [40]), and almost 10-fold higher than that determined for *Thermus thermophilus* with the NADH/coenzyme Q1 substrate pair (10 μM; [41]). Based on the kinetic analyses, *Mm*NQO shows a certain preference for NADH over NADPH. Moreover, NADH can be oxidized using all ten artificial electron acceptor substrates tested, whereas NADPH is only turned over in the presence of 1,4-BQ, coenzyme Q1 and potassium ferricyanide. These results are similar with what has been reported for wild-type *E. coli* NDH-1 [4], and to some extent also for *Corynebacter glutamicum* NADH:quinone oxidoreductase, which show higher affinity and turnover rates for NADH using coenzyme Q1 compared with NADPH [42].

Stability of *Mm*NQO activity was assessed at different pH values (2.5–10.0) using NADH and 1,4-BQ as electron donor and acceptor substrates, respectively. The enzyme was active in the pH range 4.5–8.5 with an optimum at 8.0 (Supplementary Figure S7).

### Crystal structure of *Mm*NQO

**Figure 1:**
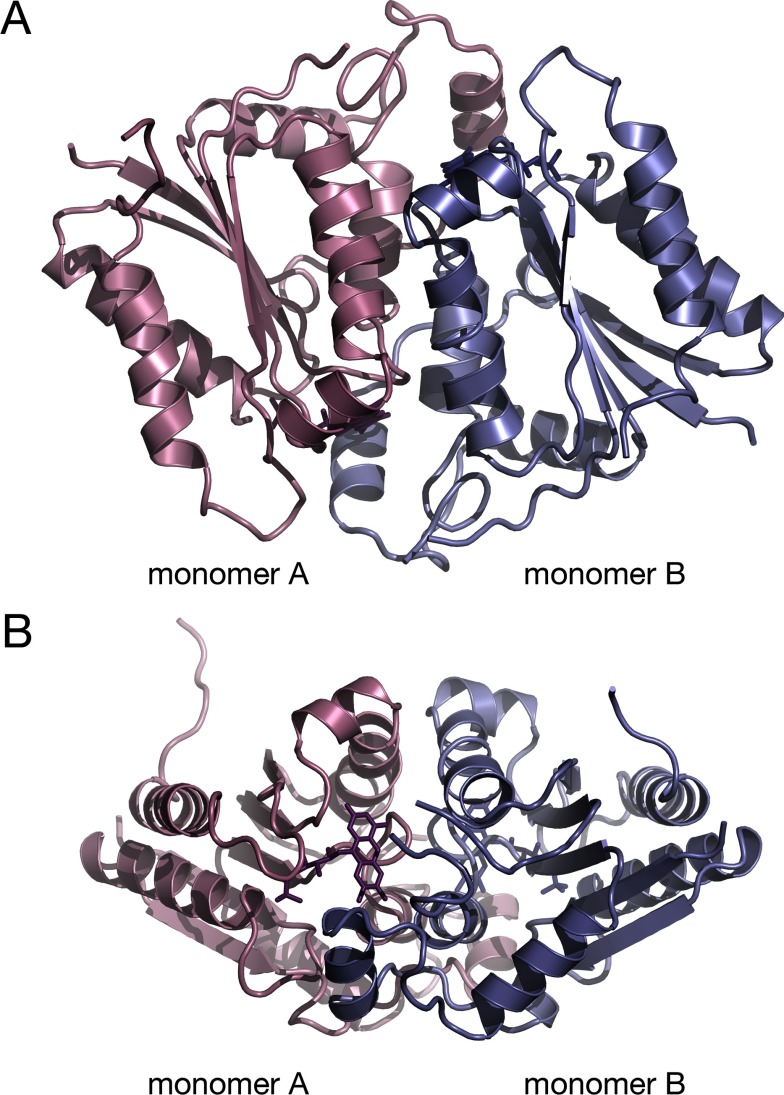
Overall fold of *Mm*NQO (**A**) The flavodoxin-like fold of monomers A and B in the A/B dimer are coloured pink and blue, respectively. The FMN molecules are shown as stick models in darker colours. (**B**) Same as in panel (**A**) but rotated 180°.

**Figure 2:**
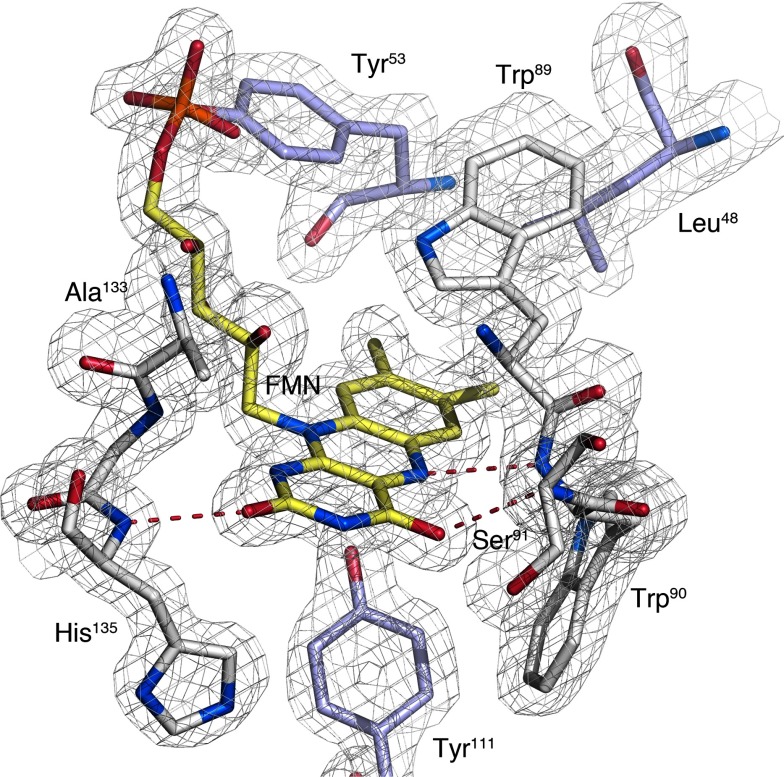
FMN-binding pocket in the monomer of *Mm*NQO The flavin-binding pocket in monomer A (white). The aromatic indole rings of Trp^89^ and Trp^90^ pack against the flavin ring (yellow). Two tyrosine and one leucine residue from the B monomer (blue) are shown.

The crystal structure of *Mm*NQO features a homodimeric enzyme where each monomer adopts the flavodoxin-like α/β-fold commonly observed for NAD(P)H:quinone oxidoreductases (Figure 1). The active sites of both monomers are situated at the dimer interface, each with a non-covalently bound FMN molecule bound approximately 8 Å below the molecule's surface. The flavin isoalloxazine ring is firmly bound by the protein (Figure 2) with its dimethylbenzene nucleus interacting with hydrophobic side chains (Trp^89^and Trp^90^ from molecule A; and Leu^48^ and Tyr^53^ from molecule B); the N_5_ atom of the central phenylenediamine ring forming a hydrogen bond to the backbone amide nitrogen of Trp^90^; and the O_2_ and O_4_ oxygen atoms in the pyrimidine of the isoalloxazine ring forming hydrogen bonds to the backbone amide groups of His^135^ and Ser^91^, respectively.

**Figure 3:**
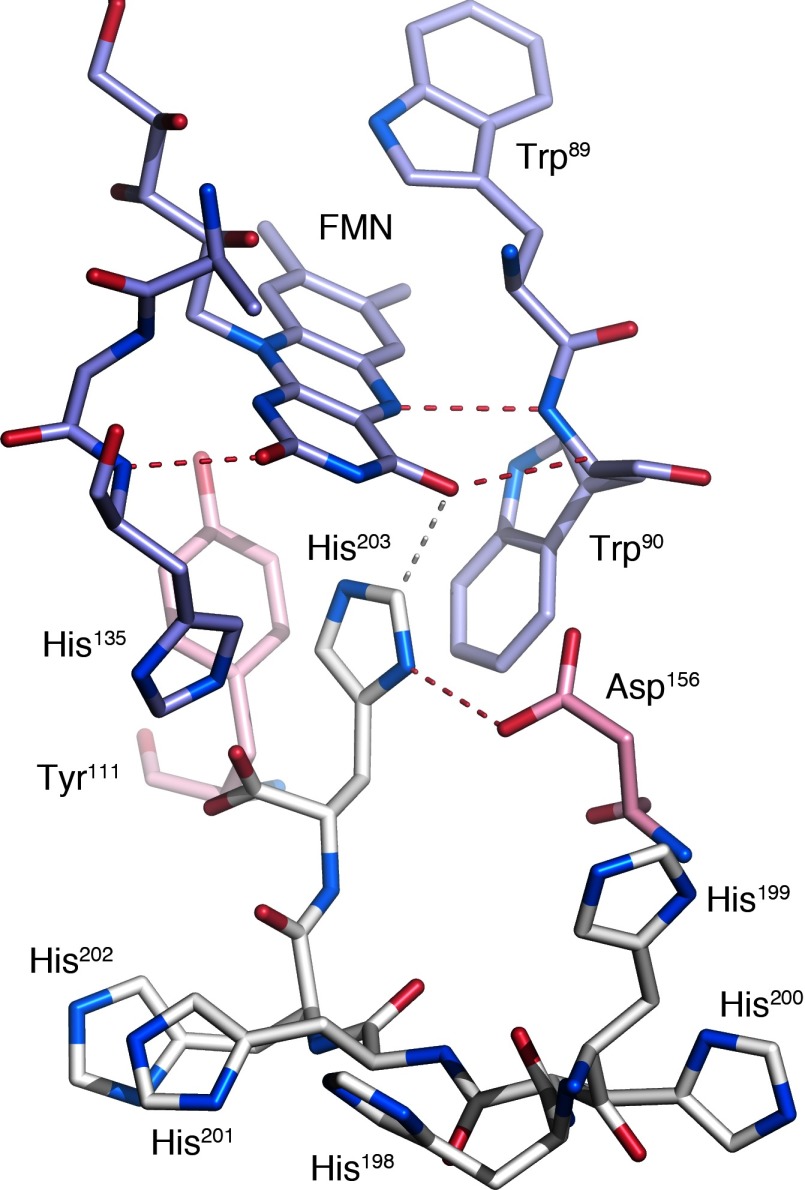
Interactions of the hexahistidine tag The hexahistidine tag (His^198^–His^203^) in molecule A (white) is interacting with the FMN-binding pocket of a crystallographically related C/D dimer (monomer C, pink; monomer D, blue). Red dashed lines represent hydrogen bonds and the white dashed line represents the separation between His^203^ CE1 and FMN O4.

There are two non-crystallographically related homodimers (A/B and C/D) in the asymmetric unit of the crystal. In each dimer, one monomer (A in the A/B dimer and C in the C/D dimer) packs such that the ultimate histidine of the C-terminal hexahistidine tag is reaching into the flavin pocket to interact with the FMN ring in a crystallographically related molecule, i.e. His^203^ in molecule A interacts with the FMN in a crystallographically related D monomer and Asp^156^ in a crystallographically related C monomer (Figure 3). This interaction places the A-His^203^ CE1 atom 3.1 Å from the O_4_ atom of D-FMN. Similarly, His^203^ in molecule C interacts with crystallographically related C-Asp^156^ and B-FMN.

### Comparison of *Mm*NQO with structurally related enzymes

Structure-based fold similarity analysis using the *DALI Lite* and *PDBeFold* servers returned three principal classes of NAD(P)H:quinone oxidoreductases: (1) modulator of drug activity B (MdaB); (2) bacterial FMN-dependent azoreductases (AzoR); and (3) mammalian FAD-dependent quinone reductases type 1 (QR1/NQR1) and 2 (QR2/NQR2). Additional protein structures occurred at lower scores, for instance, KefF of the KefC K+ efflux system, ChR (chromate reductase), EmoB of the EmoA/EmoB EDTA biodegradation system, and Trp-repressor-binding protein WrbA. The differences in sequence identity and r.m.s.d. (root-mean-square deviation) between the three principal groups are large according to the DALI analysis, typically 19–23% identity, and 2.3–2.8 Å r.m.s.d. on Cα positions. All structures display a structurally well-conserved homodimeric flavodoxin-like fold where the principal differences are noted for the precise length and conformation of mainly the dimer-interface loops delineating the flavin-binding pocket. It is thus difficult to speculate on the function of *Mm*NQO based on global sequence comparison. The azoreductases, KefF, EmoB and ChR use FMN as cofactor, whereas NQRs and MdaBs use FAD.

**Figure 4:**
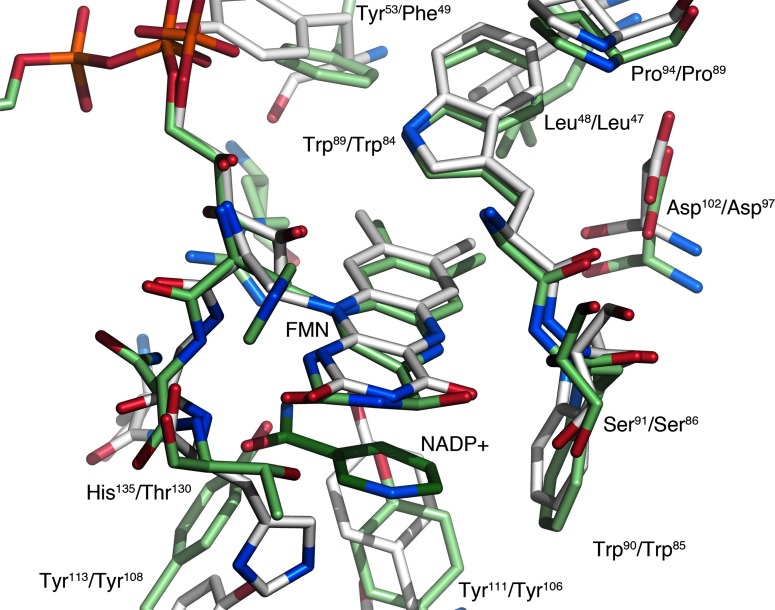
Comparison of *Mm*NQO with *Sm*MdaB Overlay of the flavin pocket in *Mm*NQO (white) and MdaB (light green) with part of the NADP+ molecule shown (dark green). Numbering of amino acids with *Mm*NQO first.

In the first group, the model of FAD-dependent MdaB from *Streptococcus mutans* scores as the most similar structure (PDB code 3LCM; [24]) and was thus used as search model in molecular replacement calculations. *S. mutans* MdaB is also the most similar structure at the local level in the immediate vicinity of the flavin. The side chains Trp^89^, Trp^90^, Tyr^111^, Tyr^113^, Asp^102^, Ser^91^ and Pro^94^ near the FMN group in *Mm*NQO are all present in *Sm*MdaB as Trp^81^, Trp^82^, Tyr^106^, Tyr^108^, Asp^97^, Ser^86^ and Pro^89^ (Figure 4). The higher local similarity in and near the flavin pocket suggests that *Mm*NQO may play a similar role, or uses similar substrates. The function of MdaBs is not fully understood, but enzymes typically provide resistance towards certain drugs [43] and defense against oxidative stress [44]. *Sm*MdaB has been confirmed to act as an FAD-dependent NADPH:quinone reductase [24]. MdaB can use menadione (vitamin K3) as electron acceptor, and binding of both NADP^+^ and menadione have been confirmed biochemically and crystallographically (PDB codes 3LCM and 4F8Y, respectively). Both ligands can also be accommodated in the active site of *Mm*NQO, but with slight adjustments relative to the *Sm*MdaB crystal structures.

**Table 2:** Kinetic constants of *Mm*NQO with NADH and NADPH (0.1–150 μM) and different electron acceptors at saturating concentrations DCPIP, dichlorophenolindophenol; n.d., not determined (the affinity was too low and no reliable kinetic constants could be determined).

NADH	Electron acceptor DCPIP	*K*_m_ (μM) 17.5	*k*_cat_ (min^−1^) 4.95	*k*_cat_/*K*_m_ (μM^−1^·min^−1^) 0.283
	Coenzyme Q10	33.7	6.86	0.204
	Potassium ferricyanide	74.8	14.3	0.191
	Fc^+^	64.8	11.2	0.173
	1,4-BQ	56.5	9.74	0.172
	Coenzyme Q1	96.8	15.6	0.161
	Diethyl-oxalacetate sodium salt	63.2	9.73	0.154
	Sodium fumarate dibasic	41.4	6.15	0.149
	DL-malic acid	102.5	14.2	0.139
	Sodium thiosulphate	257.9	19.8	0.077
**NADPH**	Coenzyme Q1	111.6	14.6	0.130
	1,4-BQ	48.1	6.0	0.125
	Potassium ferricyanide	n.d.	n.d.	n.d.

Bacterial FMN-dependent azoreductases represent the second group. Although there are similarities to *Mm*NQO in the active site, albeit lower than for MdaBs, closer inspection of existing azoreductase structures in complex with azo dyes and other ligands reveal important differences reducing the probability of *Mm*NQO being an azoreductase. Structural comparisons with azoreductases in complex with cibacron blue (PDB code 3W78), methyl red (PDB code 2V9C and 3KEG), dicoumarol (PDB code 2Z9C), balsalazide (PDB code 3LT5), nitrofurazone (PDB code 3RW6) and anthraquinone-2-sulphonate (PDB code 4C0X), reveal that neither of these ligands can be easily accommodated in the *Mm*NQO active site without steric hindrance. Cibacron blue is the most problematic ligand causing clashes with Trp^90^, His^135^ and Asp^139^ in monomer A, and Tyr^111^ and Tyr^113^ in monomer B of the dimer. For the other ligands, clashes are mainly with His^135^. Although local conformational changes may well change the precise details and relieve steric hindrance, His^135^ is very strategically positioned at one side of the FMN cofactor to restrict the substrate-binding region, a feature that does not exist in bacterial FMN azoreductases. To investigate this further, we tested two common azoreductase substrates, methyl red and dicoumarol, with *Mm*NQO. Whereas no catalytic activity could be detected with dicoumarol, catalytic constants for methyl red were *K*_m_=683 μM and *k*_cat_=6.34 min^−1^ giving a very low specificity constant of 0.009 μM^−1^·min^−1^, which is 31-fold lower than that for NADH/DCPIP (Table 2). Thus, a role of *Mm*NQO as an azoreductase is unlikely.

The third structurally related group is mammalian FAD-dependent quinone reductases. However, the active sites in mammalian NQRs have a lower local similarity to *Mm*NQO than the MdaBs. A glycine in NQR1 and NQR2 is found at the position of His^135^ in *Mm*NQO creating a more spacious active site at the pyrimidine side of the flavin ring. The only conserved side chain near the flavin ring is Trp^90^, which is occupied by Trp^105^ in NQR1 and NQR2.

**Figure 5:**
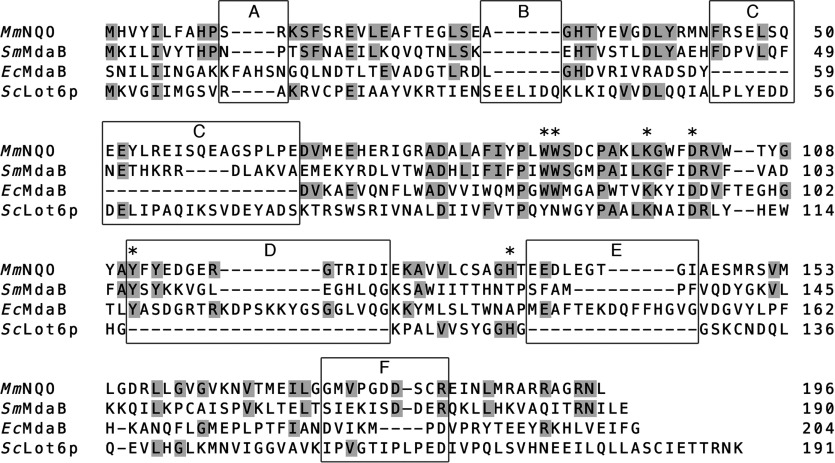
Structure-based amino-acid sequence alignment Amino-acid sequence numbering is according to that of the coordinate files: *S. mutans* MdaB (*Sm*MdaB; PDB code 3LCM), *E. coli* MdaB (*Ec*MdaB; PDB code 2B3D), *S. cerevisiae* Lot6p (*Sc*Lot6p; PDB code 1T0I). Amino acids identical to *Mm*NQO are gray-shaded. Pairwise identities based on structural alignment: *Mm*NQO-*Sm*MdaB, 23.5%; *Mm*NQO-*Sc*Lot6p, 13.8%; *Mm*NQO-*Ec*MdaB, 12.8%; *Sm*MdaB-*Sc*Lot6p, 13.3%; *Sm*MdaB-*Ec*MdaB, 16.5%; *Sc*Lot6p-*Ec*MdaB, 7.3%. Amino acids highlighted by an asterisk: the restricting His^135^ in a subunit A at the flavin A *Si*-side; Trp^89^ and Trp^90^ in subunit A flanking the flavin A *Re*- and *Si*-side, respectively; Asp^102^ and Lys^98^ in subunit A forming a salt bridge at the flavin B *Re*-side; and subunit A Tyr^111^ at the flavin B *Si*-side. The five loop regions A–F discussed in the text are boxed.

As mentioned above, the quinone reductase most similar to *Mm*NQO is the FAD-binding *Sm*MdaB. Interestingly, the *E. coli* homologue of *Sm*MdaB (*Ec*MdaB; PDB code 2B3D [45]) is strikingly different than both *Mm*NQO and *Sm*MdaB. Based on pairwise structural alignment, the amino-acid sequence identity between *Mm*NQO and *Sm*MdaB is 23.5%, whereas the identity is only 16.5% between *Sm*MdaB and *Ec*MdaB (Figure 5), revealing a more distant relationship between the two MdaBs. In addition to bacterial MdaBs, *Mm*NQO displays structural similarities to the FMN-dependent NAD(P)H:quinone oxidoreductase *Saccharomyces cerevisiae* Lot6p (former YLR011wp; PDB code 1T0I [46]). *Sc*Lot6p has been shown to act as an inducer of apoptosis [47] and is the only known quinone reductase in budding yeast [48]. The structure-based sequence identity between *Mm*NQO and *S*cLot6p is 13.8%, which is slightly higher than the pairwise identity of 13.3% between *Mm*NQO and *Ec*MdaB.

**Figure 6:**
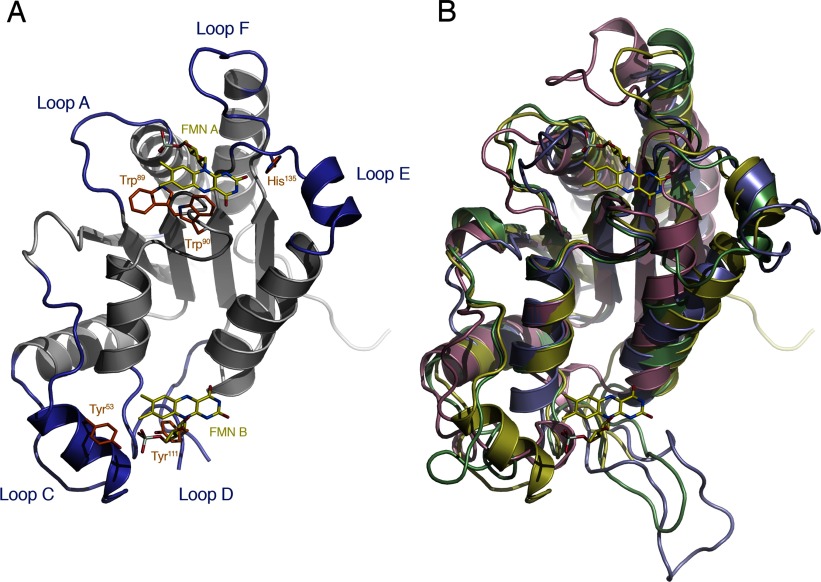
Comparison of *Mm*NQO with MdaBs and Lot6p (**A**) Ribbon drawing highlighting the six structurally variable loops (blue) in subunit A of *Mm*NQO and side chains discussed in the text (orange) that interact with the FMN molecules in subunits A and B (yellow). The view is facing the dimer interface but for clarity subunit B has been omitted. Loop D in *Mm*NQO is disordered without interpretable electron density and has not been modelled. (**B**) Structural superpositioning of *Mm*NQO (yellow), *Sm*MdaB (green; PDB code 3LCM), *Sc*Lot6p (pink; PDB code 1T0I) and *Ec*MdaB (blue; PDB code 2B3D) with the FMN molecules in *Mm*NQO shown.

When inspecting the structure-based sequence alignment for the four dimeric enzymes, six loop regions (loops A–F, Figures 5 and 6) reveal significant insertions or deletions. All loops except one (loop B) are involved in formation of the flavin- and substrate-binding region at the dimer interface. Loop A (residues 9–16 in *Mm*NQO) binds the α-phosphate moiety in FAD or FMN and coincides with the flavodoxin loop. However, the flavodoxin fingerprint (T/S)XTGXT that is normally associated with this loop in flavodoxins is not present in any of the four enzymes. In *Ec*MdaB, loop A is four residues longer compared with the other enzymes. The second loop, B, is present only in *Sc*Lot6p and is found at the face opposite to the dimer interface, and thus appears to have no role in flavin or substrate binding. Loop C (residues 44–67 in *Mm*NQO) in one subunit closes off the dimethylbenze edge of the flavin pocket in the neighbouring subunit of the dimer. Loop C is absent altogether in *Ec*MdaB, but its function appears to be partly compensated for by loop A (residues 23–28 in *Ec*MdaB) that is absent in the other enzymes. Residues 104–124 (*Mm*NQO) form an extensive D loop in *Mm*NQO and MdaBs which is located at the *Si*-side of the flavin where it creates the ‘floor’ of the substrate-binding pocket. Loop D is absent in *Sc*Lot6p leaving the FMN fully exposed to the solvent. Loop E (134–145 in *Mm*NQO) packs against the pyrimidine edge of the flavin in the same subunit. Also this loop is absent in *Sc*Lot6p. When the precise side chains that interact with the flavin ring are concerned, *Sc*Lot6p is clearly distinct from the *Mm*NQO/MdaB group (Figure 5). Interestingly however, the restricting His^135^ in *Mm*NQO is present also in *Sc*Lot6p (His^127^), but is displaced away from the flavin ring due to the absence of loop E. Loop F (173–182 in *Mm*NQO) contributes to binding of the adenosine moiety in FAD. In the two FAD-binding MdaBs, this loop is folded away to accommodate the adenosine ring whereas in the FMN-binding *Mm*NQO and *Sc*Lot6p loop F closes off around the phosphoribityl moiety of FMN.

It has been reported that during heterologous expression and purification of FAD-dependent human NQO2 in *E. coli*, a significant fraction of the enzyme binds either FMN or FAD, or a mixture thereof [49,50]. It is possible that the FMN bound to *E. coli*-expressed *Mm*NQO may not represent the physiologically relevant co-factor. To evaluate the possible preference for FMN or FAD by *Mm*NQO, a detailed comparison with the FAD-binding *Sm*MdaB provides some information. At the sequence level, the region responsible for binding the adenosine part of FAD in *Sm*MdaB is overall very similar in *Mm*NQO, i.e. loop A (residues 9–15) and loop F (residues 173–182), including an important interaction offered by His^9^ to the FMN α-phosphate group. An additional interaction to the monophosphate is provided by Tyr^53^ in *Mm*NQO, which is absent in *Sm*MdaB. Although the overall sequence similarity of loops A and F is relatively high between the two enzymes, the conformation of loop F is fundamentally different. In the FMN-bound state of *Mm*NQO, loop F is folded *around* the monophosphate moiety whereas in the FAD-bound state of *Sm*MdaB, the F-loop is folded *away* to accommodate the adenosine ring. Whether this conformational difference reflects true differences in flavin co-factor specificity is not possible to deduce from the structure alone, but it should be emphasized that the density for the FMN is excellent and the existing interactions appear very favourable. *Sc*Lot6p also binds FMN, and as observed for *Mm*NQO, loop F adopts a conformation that closes around the monophosphate group, which contrasts to the more open conformation observed for the FAD-binding enzymes *Sm*MdaB and *Ec*MdaB. Conceivably, loop F evolved an inherent ability to bind both FMN and FAD to allow either co-factor to be used by this group of enzymes.

### Conclusions

The function of *Mm*NQO inferred from structural similarity leans towards an MdaB-type activity, however it should be emphasized that the overall sequence identity is low, and r.m.s.d. values high. With respect to *in vitro* kinetics and structure, *Mm*NQO shows similarities to several quinone reductase members such as MdaB, Lot6p, and NQO2. Structural comparisons are insufficient to conclusively answer whether *Mm*NQO prefers FMN or FAD, and whether NADH or NADPH serves as coenzyme. Based on structural and biochemical data, we suggest that FMN and NADH are preferred, which justifies that *Mm*NQO is annotated as an FMN-dependent NADH:quinone oxidoreductase. The biological function of *Mm*NQO is yet to be elucidated, but from the data presented here it may constitute an archaeal counterpart of the modulator of drug activity B in bacteria. The NADH:quinone oxidoreductase activity of *Mm*NQO and its localization to the cytoplasm suggest that it may be a useful tool for applications where *E. coli* cells require a high-energy metabolism and increased capacity of NADH regeneration, e.g. recombinant protein production processes. Hitherto, attempts to eliminate overflow metabolism in *E. coli* by overexpressing NADH:quinone oxidoreductases have been unsuccessful mainly due to the stress associated with overexpression of large integral membrane complexes. The comparably small size of soluble *Mm*NQO makes it a potentially useful enzyme for regeneration of excessive NAD^+^ in bacterial hosts.
